# Novel mechanism of immune evasion mediated by tumor‐associated macrophages in esophageal squamous cell carcinoma

**DOI:** 10.1111/1759-7714.13549

**Published:** 2020-07-19

**Authors:** Fei‐Yu Diao

**Affiliations:** ^1^ Department of General Surgery Sun Yat‐Sen Memorial Hospital, Sun Yat‐Sen University Guangzhou People's Republic of China

Among all cancers worldwide, esophageal cancer was ranked seventh as the most commonly diagnosed cancer and sixth as the commonest cause of cancer‐related deaths in 2019.^1^ Esophageal squamous cell carcinoma (ESCC) accounts for the vast majority of all esophageal cancer cases.^2^ Compared with the rest of the world, China has the highest incidence of ESCC. Around half of the global ESCC cases occur in China.^3^ Due to the low rate of early diagnosis and treatment, the five‐year survival rate of ESCC remains at a very low level. Therefore, it is an urgent task to better understand the initiation and progression of ESCC and develop novel, preventive and therapeutic strategies against ESCC.

In the past, studies on cancer initiation and progression mainly focused on the identification and characterization of (epi)genetic alterations within the tumor cells. However, findings from these studies have not been well translated from bench to bedside.^4^, ^5^ Currently, there is mounting evidence indicating that the tumor microenvironment (TME), especially tumor‐associated immune cells, is equally important for the initiation and progression of cancer.^6^ Therapeutic strategies that target tumor‐associated immune cells, rather than tumor cells, have shown significant efficacy in multiple cancer types.^7^, ^8^

Previous studies have identified several tumor‐associated immune cells and investigated the immune landscape of ESCC.^9^ Many preclinical or clinical studies on immunotherapy targeting such immune cells have been performed in ESCC in recent years and some of them have yielded promising results.^10^ These observations indicated that targeting tumor‐associated immune cells holds untold prospects for possible applications in the treatment of ESCC. A better understanding of the molecular mechanisms by which tumor‐associated immune cells promote tumor initiation and progression can help to develop better immunotherapy strategies for ESCC.

In a study recently published in *Molecular Cancer*, “CCL2/CCR2 axis recruits tumor‐associated macrophages to induce immune evasion through PD‐1 signaling in esophageal carcinogenesis,”^11^ the research team led by Professor Xudong Jia of the China National Center for Food Safety Risk Assessment (Beijing, China) analyzed the transcriptional profiling of an ESCC rat model to explore the underlying mechanism of tumor‐associated immune cells in ESCC initiation. They found that the recruitment of immune cells regulated by chemokines was markedly changed in the ESCC rat model. The expression of C‐C motif chemokine ligand 2 (CCL2) was upregulated continuously during esophageal carcinogenesis. CCL2 is one of the leading chemokines in TME. CCL2/C‐C motif chemokine receptor 2 (CCR2) signaling axis has been reported to play a major role in recruiting tumor‐associated macrophages (TAMs), which was subsequently found to contribute to cancer progression.^12^ However, the tumor‐promoting mechanism of TAMs and the involvement of CCL2/CCR2 signaling axis in ESCC initiation has not yet been investigated. Thus, the authors attempted to explore the role of CCL2/CCR2 signaling axis in TAMs recruitment and activation during human esophageal carcinogenesis. First, they determined CCL2 distribution in ESCC tissue microarray and compared the number of TAMs (CD68‐positive cells) with CCL2 expression. Their results showed that CCL2 expression levels in ESCC tissues were positively correlated with the number of TAMs. Further, multivariate Cox analysis suggested that CCL2 upregulation and an increase in the number of TAMs were associated with poor prognoses in patients with ESCC. In an ESCC rat model, the authors observed that CCL2 was associated with TAM accumulation in esophageal carcinogenesis induced by nitrosamine. In addition, TAM accumulation and esophageal carcinogenesis were inhibited when the CCL2/CCR2 axis was blocked. In order to explore the underlying molecular mechanism of TAM infiltration and TAM‐mediated carcinogenesis, the authors analyzed the tumors harvested from a CCR2−/− mouse model by RNA‐sequencing. They found that the programmed death‐1 (PD‐1) signaling pathway in T cells was significantly suppressed by CCR2 in mouse models. In other words, TAMs mediated the antitumor effector T cell depletion and thereby facilitated ESCC cell evasion via the PD‐1 signaling pathway. Furthermore, gene set enrichment analysis using The Cancer Genome Atlas (TCGA) data indicated a connection between CCL2/CCR2 signaling axis and PD‐1 signaling in ESCC. These results suggested that the CCL2/CCR2 axis could promote ESCC initiation by inducing immune escape which was mediated by TAMs through the PD‐1 signaling pathway. Previous studies have shown that TAMs impacted tumor initiation and progression by generating TAMs subtypes with diverse properties.^13^ Similar evidence was also observed in this study. As compared with the CCL2+/+ mice, CCL2−/− mice had fewer M2‐type of TAMs but more antitumor effector T cells. Therefore, the authors further examined the expression of programmed death‐ligand 1 (PD‐L1) and programmed death‐ligand 2 (PD‐L2) in M1‐ and M2‐type TAMs to evaluate the impact of TAMs polarization on PD‐1 signaling. They found that M2 polarization of TAMs could upregulate PD‐L2 expression to enhance immunosuppression in ESCC.

The functional mechanisms of TAMs have been characterized in various cancer types. Additionally, TAM‐based anticancer therapies have shown remarkable potential to synergize with chemotherapy^14^ and immunotherapy.^7^ However, their functional mechanisms in ESCC were still unclear. In the present study, the authors showed the critical role of TAMs in ESCC initiation by focusing on their recruitment and polarization. Different from previous studies which reported that CCL2 could directly impact ESCC cells,^15^ this study demonstrated that CCL2 played a role in bridging tumor cells with TAM‐mediated immune response during the initiation of ESCC. Furthermore, a discrepant expression of PD‐L2 in the two subtypes of TAM was also observed in this study. This discrepancy was responsible for M2‐type TAM‐mediated esophageal carcinogenesis. Therefore, molecules that regulate the M2‐polarization of TAMs can be used as new immune checkpoint targets for ESCC.

## Disclosure

The author declares no competing interests.
